# Prevalence of Intestinal Parasites among Rural Residents of Takestan in North-West of Iran

**Published:** 2019

**Authors:** Khadijeh TAHERKHANI, Ameneh BARIKANI, Mojtaba SHAHNAZI, Mehrzad SARAEI

**Affiliations:** 1. Department of Medical Parasitology and Mycology, School of Medicine, Qazvin University of Medical Sciences, Qazvin, Iran; 2. Children Growth Research Center, Qazvin University of Medical Sciences, Qazvin, Iran; 3. Cellular and Molecular Research Center, Qazvin University of Medical Sciences, Qazvin, Iran

**Keywords:** Prevalence, Intestinal parasites, Human, Iran

## Abstract

**Background:**

Intestinal parasites are one of the health challenges in developing countries. Decreasing the prevalence of intestinal parasitic infections (IPIs) is one of the main aims of health services in these countries. This study was designed to determine the current status of IPIs in rural residents of Takestan a town located in North West of Iran.

**Methods:**

A total of 2280 rural residents of Takestan were randomly selected. Data were collected through questionnaire by interviews and laboratory findings obtained by microscopic examination of stool sample including wet smear and formalin ethyl-acetate concentration. A *P*<0.05 was considered significant, statistically.

**Results:**

In total, 8.7% (199/2280) of participants were positive for at least one intestinal parasite. The prevalence of polyparasitism was 0.7% in study population. *Hymenolepis nana* was the only helminthic infection which was detected (1/2280). *Blastocystis*, *Entamoeba coli*, and *Giardia lamblia* were the most common IPIs with prevalence of 3.6%, 2.9%, and 1.6%, respectively. Statistically, the prevalence of IPIs showed significant differences among villages (*P*<0.01) and age groups (*P*<0.001), and also habit of eating raw vegetables (*P*<0.005), whereas, the difference was insignificant in terms of sex, education level, and occupation.

**Conclusion:**

The prevalence of IPIs in rural residents of the study area is considerably low and this reduction was very impressive about helminthic infections.

## Introduction

The intestinal parasitic infections (IPIs) are still a major health problem in many developing countries. High prevalence rates for intestinal parasitic infections are reported from rural inhabitants of Malaysia (73.2%) (1), rural schoolchildren in Mexico (57.0%) (2), primary school children in Ethiopia (81.0%) (3), and schoolchildren in Tripoli, Lebanon (85.0%) (4).

Iran, located in West Asia is also a developing country but with a different pattern in recent years compared to most of these countries. In the past, i.e. over four decades ago, IPIs especially soil-transmitted helminthes were enormously common in Iran, particularly in the north, northwest, and western parts of the country. Hence, based on several reports, it was estimated that at least 50% of people were infected with pathogenic intestinal parasites (5–8). At present, the prevalence of parasites has dramatically decreased in Iran, for example, 4.7% of 13915 residents of Karaj (9), 11.9% of 816 bakery workers in Khorramabad (10), 15.5% of the 1041 food handlers in Sari (11), and 10.4% of 1021 food handlers in Shiraz (12) were positive for IPIs. Rarely, a prevalence rate higher than 30% has been reported from Iran in the recent years (13,14).

The previous studies on prevalence of IPIs in Iran have mostly focused on schoolchildren (14), occupations (10–12), and people who were referred to a laboratory center (15). Community-based studies on general population provide more accurate description about the current status of these infections in a community, unfortunately, the number of such studies reported from Iran is limited and this was the reason why the present study was performed. On the other hand, there was no previous report about the status of IPIs in the study area, therefore it was the main reason for performing the present study.

## Methods

### The study area

**Fig. 1:**
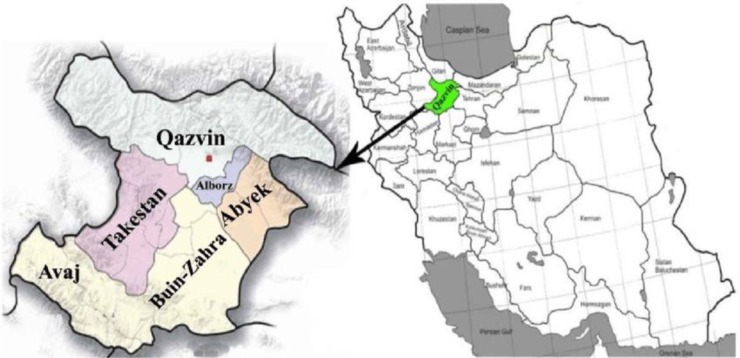
The geographical location of the study area. Map of Iran (Right) and map of Qazvin province (left). Takestan is located in west of this province

This study was conducted in villages of Takestan located in Qazvin province, northwest of Iran (Fig. 1). It has a cold semi-arid climate. In 2014, annual rainfall was about 200 mm with relative humidity of at least 20% in September and the utmost humidity of 58% in December.

### Population, sampling, and ethical considerations

Initially, this project was approved by the Ethics Committee of Qazvin University of Medical Sciences (IR.QUMS.REC.1394.111).

The present study was carried out on rural residents of Takestan. The sample size was estimated at 2280 specimens (with counting P=1.0%, d=5.0%, level of confidence=95.0%). Multistage cluster method was used for sampling. This cross-sectional epidemiological study was carried out from May to October 2015. Overall, 26 villages were selected. The sampling in each village was conducted in collaboration with rural health houses as follows:

In the beginning, the study objectives and procedures were explained verbally for all participants, and then, handouts containing information about intestinal parasitic diseases, the aims and procedures of the study, obligation-free participation, availability of lab tests at no cost, and the phone number of project manager. Written informed consents were taken from all volunteers. The participants filled a questionnaire containing individual, socio-demographic, and health information. The fecal samples were collected into clean and dry plastic containers from May to October 2016. The results for all samples were sent to the participants and the subjects who were infected with pathogenic intestinal parasites were referred to clinicians for anti-parasitic treatments.

### Stool examinations

One fresh fecal sample from each participant was examined. All samples were initially examined through wet smears prepared with normal saline (0.85% NaCl solution) and Lugol’s Iodine solution. The negative samples for parasitic agents were re-examined by using formalin ethyl-acetate concentration method. The Gomori’s trichrome staining technique was used as a confirmatory test for detecting the *Blastocystis*, intestinal amoebae and flagellates observed in wet smears or concentration method. In addition, we used a modified Ziehl-Neelsen acid-fast technique to detect the oocysts of *cryptosporidium* spp. and *cyclospora* spp. in stool samples. The prepared slides were observed under light microscope at ×10, ×40, and ×100 objective magnification (16).

### Statistical analysis

Data were analyzed using the Statistical Package for the Social Sciences (SPSS) (SPSS Inc., Chicago, IL, USA) software version 19. The absolute and relative frequencies were used to describe the prevalence of IPIs according to sex, age, education, occupation, habit of raw vegetable consumption, water supply, and symptoms. Pearson’s Chi-square (Chi2) test was used for showing relationships between the prevalence of intestinal parasites and other variables. Odds ratio (OR) were computed to measure the strength of association. A *P* value <0.05 was considered significant, statistically.

## Results

Overall, 8.7% (199/2280) of the participants were infected with at least one species of intestinal parasite. Mixed infection (infection with two or three parasites) was 0.7% (Table 1). Regardless of *Blastocystis*, 1.6% of subjects were infected with pathogenic intestinal parasites, including *Giardia lamblia* and *Hymenolepis nana*. In our study, *Blastocystis* was not considered pathogenic.

**Table 1:** Prevalence of intestinal parasitic infections among rural residents of Takestan county,

***Intestinal Parasite***	***N***	***%***
Non-infected	2081	91.3
*Blastocystis* sp*.*	70	3.1
*Entamoeba coli*	58	2.5
*Giardia lamblia*	34	1.5
*Endolimax nana*	18	0.8
*Iodeamoeba butchlii*	2	0.08
*Hymenolepis nana*	1	0.04
*Blastocystis* sp.+*Entamoeba coli*	4	0.2
*Blastocystis* sp.+*Iodeamoeba butchlii*	2	0.08
*Giardia lamblia*+*Entamoeba coli*	2	0.08
*Blastocystis* sp.+*Entamoeba hartmani*	1	0.04
*Endolimax nana*+*Dientamoeba fregilis*	1	0.04
*Endolimax nana*+ *Entamoeba coli*	1	0.04
*Entamoeba coli*+ *Iodeamoeba butchlii*	1	0.04
*Blastocystis* sp.+*Endolimax nana*+*Dientamoeba fregilis*	1	0.04
*Blastocystis* sp.+ *Endolimax nana* + *Iodeamoeba butchlii*	1	0.04
*Blastocystis* sp.+ *Entamoeba coli* + *Iodeamoeba butchlii*	1	0.04
*Blastocystis* sp.+*Endolimax nana*	1	0.04
Total	2280	100

North-West of Iran (n=2280)

The prevalence of IPIs among rural districts showed significant differences (*P*<0.01). In terms of literacy level, the IPIs showed insignificant difference among participants with different educational levels (Table 2).

**Table 2:** Univariate analysis of risk factors associated with intestinal parasitic infections among rural residents of Takestan county, North-West of Iran (n=2280)

***Variables***	***Positive n (%)***	***Negative n (%)***	***OR***	***CI_95%_***		**P*-value***

				**Lower**	**Upper**	
**Sex**						0.453
Female	111(9.1)	1103(90.9)	Reference			
Male	88(8.3)	978 (91.7)	1.118	0.835	1.499	0.453
**Educational levels (Pre-primary age)**	35 (8.6)	372(91.4)	Reference			0.71
Illiterate	76 (10)	684 (90)	1.181	0.776	1.797	0.465
Primary	47(11.2)	373(88.8)	1.339	0.845	2.123	0.245
Secondary	12(8.2)	135(91.8)	0.945	0.476	1.873	1.00
High schools	28(914)	271(90.6)	1.098	0.652	1.849	0.790
Collage and above						
**Habits of raw vegetable consumption**						0.005
No + Rarely	5 (2.3)	211 (97.7)	Reference			
Daily	63 (10)	567 (90)	4.689	1.861	11.816	<0.001
At least once a week	110 (9.2)	1086(90.8)	4.274	1.724	10.601	<0.001
At least once a month	21 (8.8)	217 (91.2)	4.084	1.512	11.030	<0.01
**Occupation(≥7 years old)**						0.37
Workless & others	14(6.2)	212(93.8)	Reference			
Worker	27(11.5)	206(88.5)	0.499	0.254	0.978	0.045
Farmer	28(11.1)	225(88.9)	0.531	0.272	1.035	0.07
Gov’t employer	3(6.8)	41(93.2)	0.881	0.242	3.205	0.7
School student	32(8.5)	344(91.5)	0.703	0.361	1.367	0.3
University student	2(11.8)	15(88.2)	2.019	0.419	9.719	0.3
Housekeeper	92(10.4)	796(89.6)	0.569	0.318	1.019	0.05
**Age (yr)**						<0.001
≤ 9	10 (2.7)	364 (97.3)	Reference			
10–19	29 (9.4)	278 (90.6)	3.797	1.820	7.923	<0.001
20–29	36 (9.1)	359 (90.9)	3.650	1.785	7.466	<0.001
30–39	53 (12.5)	371 (87.5)	5.200	2.605	10378	<0.001
40–49	19 (6.2)	289 (93.8)	2.393	1.096	5.226	0.03
50–59	28 (13.3)	182 (86.7)	5.600	2.662	11.780	<0.001
60–69	18 (12.2)	130 (87.8)	5.040	2.268	11.200	<0.001
70–79	5 (5.5)	86 (94.5)	2.116	0.705	6.351	0.172
≥80	1 (4.3)	22 (95.7)	1.655	0.203	13.514	0.635

n=number; OR=odds ratio; Reference= the subgroup was considered as baseline

However, the highest prevalence rates (11.2%) were observed in secondary school students. Also, there were significant differences between the prevalence found for IPIs and the habits of raw vegetables consumption in the participants (*P*<0.005) with lowest prevalence (2.3%) rate in the subjects who either did not eat raw vegetables or just rarely consumed raw vegetables. The difference in prevalence rates of IPIs was significant among the study age groups (*P*<0.001). The prevalence of infections in the subjects <30 years was significantly lower than that found in the participants who were ≥30 years (*P*<0.03). The prevalence of IPIs in males (8.3%) and females (9.1%) showed no significant difference. Univariate analysis showed insignificant difference among the occupations.

## Discussion

The results of this study indicated that the prevalence rate of IPIs, especially those of helminthic infections, have dramatically reduced among the rural residents of Takestan. This finding is in agreement with the study by Sadeghi et al who reported a prevalence 5.92% in patients suspected of having IPIs and referred to a clinical laboratory in city of Eghbalieh located in Qazvin province (15). The results of studies from other areas of Iran indicate that the prevalence of IPIs, in particular intestinal helminthic infections (IHIs) have significantly reduced in the country in recent decades. In tribes of Chelgerd, located in southwest of Iran, the prevalence of IHIs in inhabitants and was 0.9% (6/655) (17). *Enterobius vermicularis* (2.2%), *Trichostrongylus* sp. (2.1%), and *Strongyloides stercoralis* (1.6%) were the IHIs reported in the schoolchildren of Sari, northern Iran (13). The prevalence of soil transmitted helminthes (*Ascaris lumbricoides*, hookworms, *Trichuris trichiura*) was estimated to be less than 1% in Iran (18).

In our study, 8.7% of rural inhabitants were positive for intestinal parasites considerably lower than that found in similar population in Brazil (64.3%) (19), Bolivian Chaco (86%) (20), Indonesia (95.5 %) (21), and Ethiopia (50.2%) (22). The prevalence of IPIs in our study was even lower than that found in Boyer-Ahmad district, Southwestern Iran 37.5%, the only population-based study performed in recent decade in rural areas of Iran (23). In the present study, similar to most studies reported from Iran in the recent years, *Blastocystis* sp. and *G. lamblia* were the most common intestinal parasites (10–13, 17). Higher prevalence rates of these parasites may be due to possible zoonotic transmission of these agents.

It seems that the sharp decline in the prevalence of IPIs could be linked to the synergistic effects of the following factors:

Establishment and expansion of primary health care (PHC) network: The most basic unit of the PHC is named as health house and is usually the most accessible health facility to the rural population. It seems that the services provided by health houses are among the most important factors influencing the reduced prevalence of IPIs in Iran.

Reduction of surface soil contamination with human excreta: The contamination of surface soil with human excreta is considered as one of the most affecting factors in the transmission of IPIs to human. In the more distant past when the chemical fertilizers were not available for strengthening the farmlands and in addition, the farmers did not yet believe the usefulness of the chemical fertilizers, the human excreta was traditionally used as fertilizer in some areas of Iran. Therefore, the high prevalence of *A. lumbricoides* in some areas of the country, especially Isfahan province has been attributed to the widespread use of human excreta as fertilizer in farmlands (5). In recent decades, this insanitary habit has almost been given up in Iran.

Promotion of literacy and health awareness: In the past, i.e. over four decades ago, the level of literacy and health awareness among rural populations were so low and the majority of people were illiterate and unaware of transmission routs of IPIs. At present, literacy has dramatically increased among rural residents of Iran. On the other hand, health knowledge of people, including illiterates has considerably improved in the recent years.

Easy access to anti-intestinal parasitic drugs: Prior to the establishment of health houses, anti-parasitic drugs were not easily available for rural residents of Iran, particularly for those who live in remote and deprived villages. At present, these drugs are easily available for all people through health houses, even in the most remote areas of the country.

Safe water supply and expansion of drinking water pipeline: Before the revolution of 1979, most rural residents in Iran were deprived of piped drinking water. Nowadays, the piped water is the main source of drinking water in most villages of the country. In the present study, the source of drinking water in all villages under study was piped water extracted from deep wells.

At present, it seems that the most important risk factors for IPIs in Iran are consumption of raw vegetables and close contact to animals.

## Conclusion

The prevalence of human intestinal parasites, in particular the intestinal helminthes, has remarkably reduced in Iran, so that they are no longer considered as a main health problem in most areas of the country within the recent years.
